# An Abortionist’s “Diploma”

**Published:** 1871-09

**Authors:** 


### An Abortionist's "Diploma."

The report in the New York *Tribune* concerning a recent terrible case of homicide at the hands of an abortionist, says:

Within a few weeks a villain has been arrested in New York city for perpetrating an abortion on a woman which killed her. He put her corpse into a trunk and shipped it to Chicago. This rascal, who claims to be a doctor of medicine, formerly kept a lager beer saloon in Chatham street, and about a year ago procured a diploma from a spurious medical college in Philadelphia for $100, since which time he has been practising his profession of abortionist with wonderful success. Besides a house in Amity place, he had several others in different parts of the city and a numerous corps of assistants. This is one of the results of diploma peddling.

"Rosenzweig, the abortionist, is a large man, about 5 feet 3 inches in height, and with a piercing blue eye. In answer to the questions of reporters, he said that he obtained his diploma from a medical college in Philadelphia, paying for it $40, and that his diploma is from a Philadelphia Eclectic College."

We would be glad to get a sight of the "diplomas" of all these professed abortionists in our cities. There is little doubt that those that have any diplomas at all nearly all belong to the "$40 C. O. D." class.

We take the above from the Medical and Surgical Reporter, and have only to add that this Diploma was obtained of the firm we gave a gratuitous advertisement not long since in the remarkable correspondence recently sent us by Dr. C. P. Chamberlain, of Canisteo, N. Y. It is quite time that these swindlers were "shut up."

